# Concentration-dependent photothermal conversion efficiency of gold nanoparticles under near-infrared laser and broadband irradiation

**DOI:** 10.3762/bjnano.14.20

**Published:** 2023-02-06

**Authors:** Vikas, Raj Kumar, Sanjeev Soni

**Affiliations:** 1 Academy of Scientific and Innovative Research (AcSIR), Ghaziabad-201002, India; 2 Biomedical Applications Group, CSIR ­ Central Scientific Instruments Organisation, Sector-30C, Chandigarh-160030, India; 3 Micro and Nano Optics Centre, CSIR ­ Central Scientific Instruments Organisation, Sector-30C, Chandigarh-160030, India

**Keywords:** broadband irradiation, gold nanoparticles, laser, near-infrared, photothermal conversion efficiency, plasmonics

## Abstract

The photothermal conversion efficiency of gold different nanoparticles (GNPs) in different concentrations (1.25–20 µg/mL) and at different irradiation intensities of near-infrared (NIR) broadband and NIR laser irradiation is evaluated. Results show that for a concentration of 20.0 µg/mL, 40 nm gold nanospheres, 25 × 47 nm gold nanorods (GNRs), and 10 × 41 nm GNRs show a 4–110% higher photothermal conversion efficiency under NIR broadband irradiation than under NIR laser irradiation. Broadband irradiation seems suitable to attain higher efficiencies for the nanoparticles whose absorption wavelength is different from the irradiation wavelength. Lower concentrations (1.25–5 µg/mL) of such nanoparticles show 2–3 times higher efficiency under NIR broadband irradiation. For GNRs of sizes 10 × 38 nm and 10 × 41 nm, the different concentrations show almost equal efficiencies for NIR laser and broadband irradiation. On increasing the irradiation power from 0.3 to 0.5 W, for 10 × 41 nm GNRs in the concentration range of 2.5–20.0 µg/mL, NIR laser irradiation results in 5–32% higher efficiencies, while NIR broadband irradiation leads to a 6–11% increase in efficiency. Under NIR laser irradiation, the photothermal conversion efficiency increases with an increase in optical power. The findings will facilitate the selection of nanoparticle concentrations, irradiation source, and irradiation power for a variety of plasmonic photothermal applications.

## Introduction

Plasmonic photothermal properties of gold nanoparticles (GNPs) are useful for a variety of applications including those in biomedicine, such as drug delivery, therapeutics, and diagnostics [1–5]. The interaction of free electrons of gold nanoparticles with electromagnetic fields leads to oscillations of the electrons at plasmonic resonance frequencies. Nonradioactive decay of these oscillations causes the conversion of electromagnetic energy into heat [6]. Localized heat generation through GNPs under irradiation can be used for hyperthermia treatment of tumors, termed plasmonic photothermal therapy (PPTT) [7–11]. The net temperature rise of a GNP-containing medium highly depends on shape and size of the GNPs, the dielectric constant of the medium, and the characteristics of irradiation source [12–17]. For biomedical applications, to achieve maximum light penetration, a light source of spectral output within 700–900 nm (NIR-I range, termed therapeutic window) is required [18–20]. Here, the photothermal conversion efficiency is an important parameter to assess the amount of heat generated through GNPs under NIR irradiation [21–22].

Temperature rise and photothermal conversion efficiency of different shapes of GNPs under laser irradiation have been reported [15,23–25]. The plasmonic resonance wavelength of nanoparticles shifts due to changes in the surrounding medium [14,17,26]. Thus, the same laser source may lose its suitability for applications such as photothermal therapeutics, photoacoustic imaging, and drug delivery. Also, the maximum absorption of the incident radiation is desired, which depends on the optical absorption characteristics of the nanoparticles. Thus, a broadband irradiation source may be useful in which the full width half maximum of the optical output can be tuned to suit the optical absorption characteristics of the nanoparticles. To the best of our knowledge, the measurement of the concentration-dependent photothermal conversion efficiency of various shapes and sizes of gold nanoparticles under broadband irradiation has not been reported to date.

In this study, the net temperature rise and the photothermal conversion efficiency of gold nanoparticles are evaluated under irradiation using a broadband light source and a near-infrared laser (for comparison). Gold nanospheres (GNSs) of 40 nm diameter and gold nanorods (GNRs) of sizes 25 × 47 nm, 10 × 38 nm, and 10 × 41 nm were examined. The photothermal conversion efficiency for these GNPs is reported for different nanoparticle concentrations and irradiation power values.

## Experimental

### Nanoparticles

Gold nanospheres of 40 nm diameter (product no. 741981) and GNRs of 25 × 47 nm size (product no. 771651) were purchased from Sigma-Aldrich. GNRs of 10 × 38 nm size (product no. A12-10-780-CTAB-DIH-1-25) and GNRs of 10 × 41 nm size (product no. A12-10-808-CTAB-DIH-1-25) were purchased from Nanopartz Inc. Suspensions with different concentrations of these GNPs were prepared by using ultrapure water (18.1 MΩ resistivity).

### Plasmonic photothermal measurements

**Figure 1:**
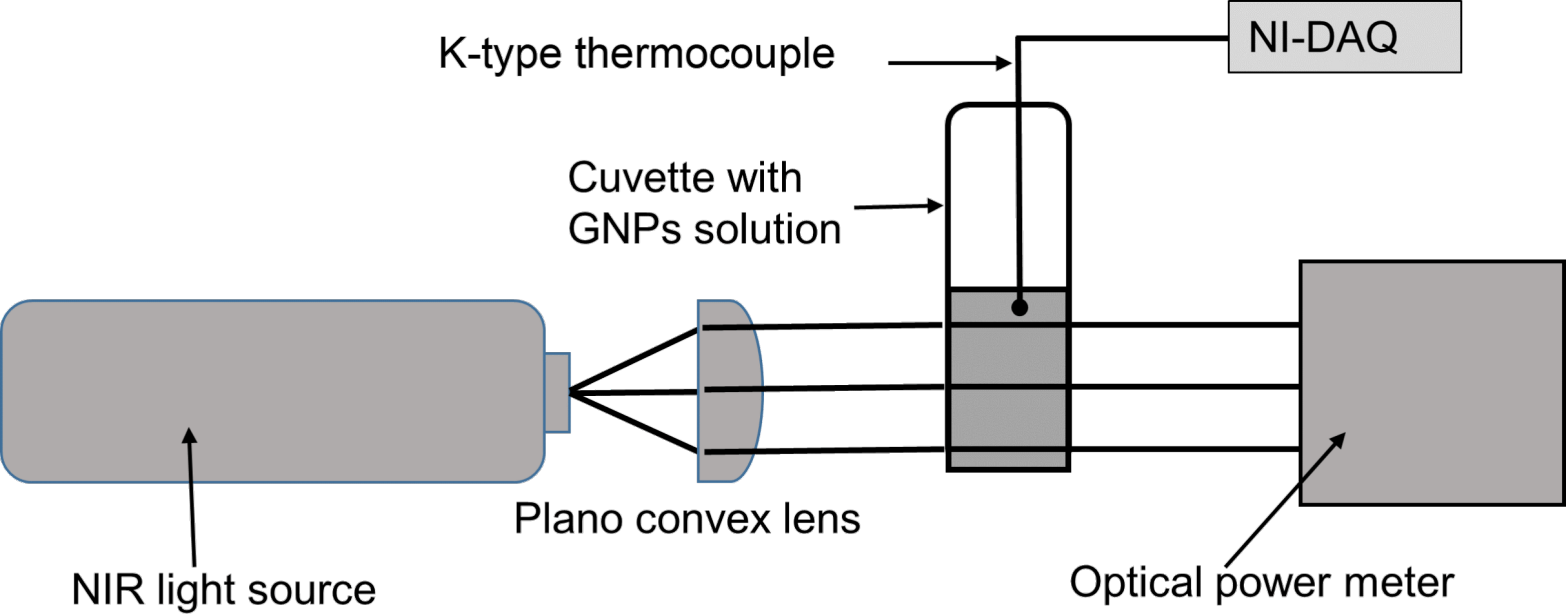
Experimental setup for the measurement of the temperature rise of GNPs suspensions under irradiation. A NIR broadband light source (754–816 nm) and a NIR diode laser (808 nm) with irradiation intensity of 0.8 W/cm^2^ are used.

The photothermal experiments were performed by adding a 1.5 mL volume of different GNP concentrations in a standard quartz cuvette of 10 mm path length. GNPs were irradiated separately by using a NIR broadband source (754–816 nm, in-house development) or an 808 nm continuous-wave diode laser (INFINITY Laser, New Age Instruments & Materials Pvt. Ltd.) with an irradiation intensity of 0.8 W/cm^2^. The incident and transmitted optical power were measured by using a digital power meter (843-R, Newport Corporation) with a thermopile detector (919P-250-35, Newport Corporation). The temperature rise of these GNP suspensions was measured by using a K-type thermocouple, and the data was acquired by using a data acquisition system (NI-myDAQ 9214, National Instruments). The schematic of the photothermal experimental setup is shown in Figure 1.

**Figure 2:**
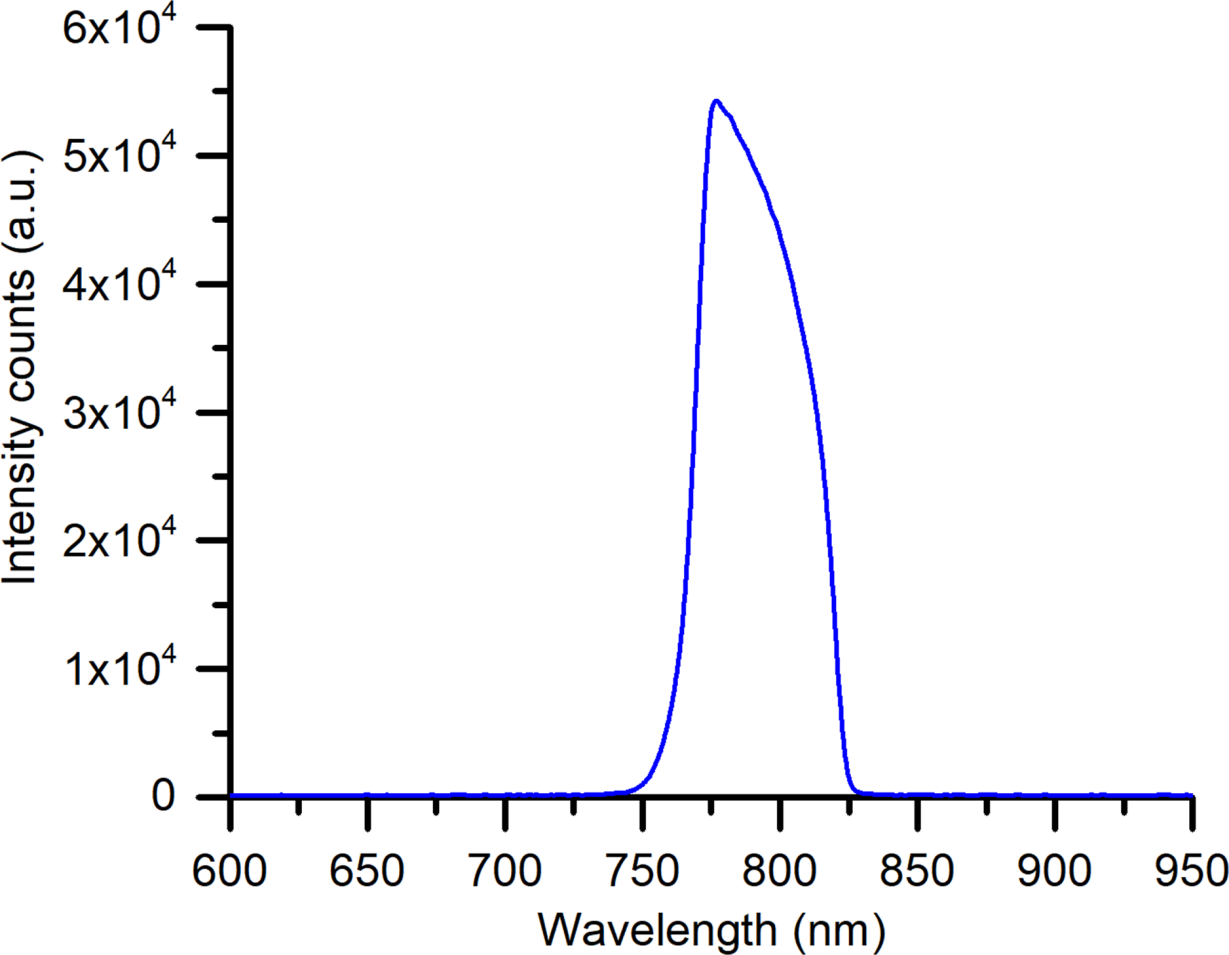
Measured spectral output of the NIR broadband light source.

The collimated input beam of 9 mm diameter was generated by using a plano convex lens of 15 mm focal length. The thermocouple tip was placed just above the path of the input beam to avoid direct irradiation of the thermocouple. During photothermal experiments, the cuvette was not sealed, and no evaporation was observed. The temperature of the GNP suspensions was recorded for a total of 1800 s (900 s with irradiation and the following 900 s without irradiation) using a K-type thermocouple (3971589 RS Components). The spectral output of the NIR broadband source was measured using a spectrometer (BLUE-Wave, StellarNet Inc) and is shown in Figure 2. The NIR broadband source exhibits a peak irradiation wavelength of 785 nm and a bandwidth of 62 nm.

**Figure 3:**
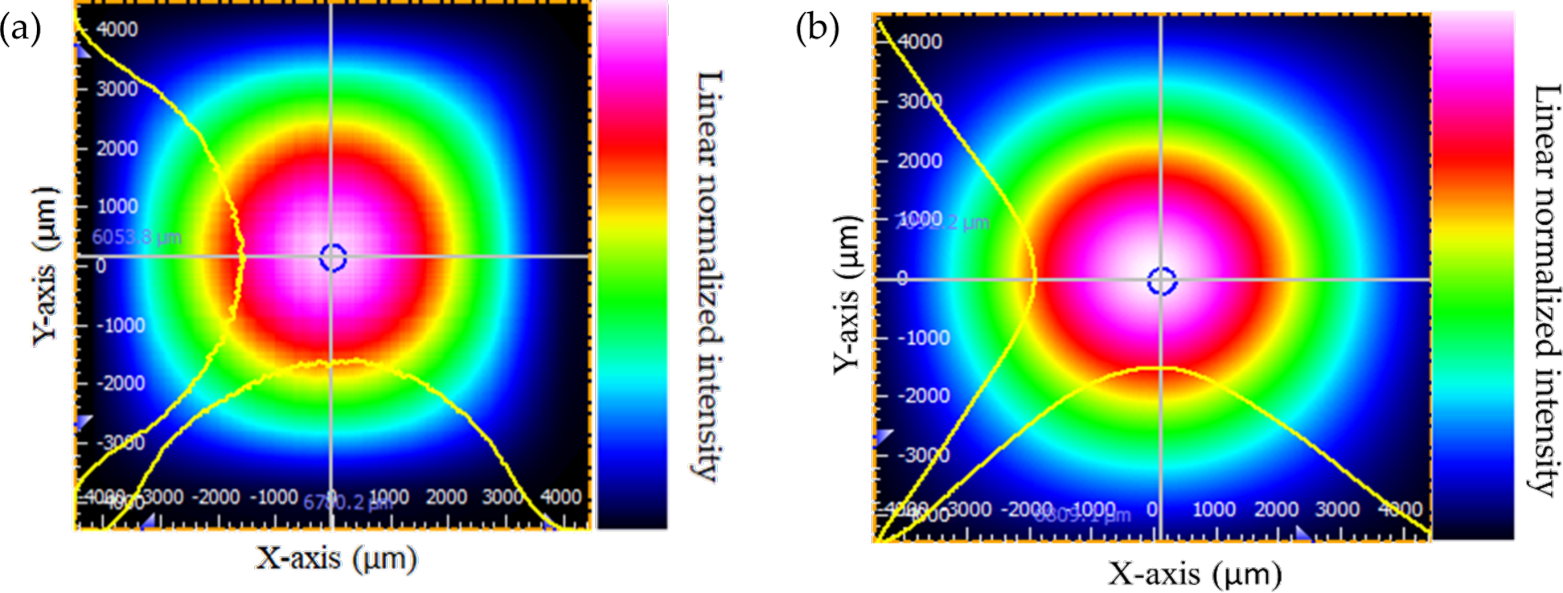
Measured beam profiles of (a) laser and (b) broadband light sources.

The *X*–*Y* axis light intensity distribution of laser and broadband sources are shown in Figure 3. The beam profiles of laser and broadband sources resemble Gaussian beam shapes as reported in our earlier article [27].

### Theory of photothermal conversion efficiency

The photothermal conversion efficiency of a GNP suspension can be analyzed by using the energy balance equation [28–29] as described in Equation 1,


[1] $$\sum_{j}C_{j}m_{j}\frac{\text{d}T}{\text{d}t}=Q_{\text{absorb}}-Q_{\text{diss}},$$

where *C_j_* and *m_j_* are, respectively, specific heat capacity and total mass of the GNP suspension, d*T* is the change of temperature of the suspension within the time interval d*t*. *Q*_absorb_ and *Q*_diss_ are, respectively, the amounts of absorbed energy and energy dissipated to the medium. The absorbed energy *Q*_absorb_ is defined as:


[2] $$Q_{\text{absorb}}=\left(P_{\text{input}}-P_{\text{trans}}\right)\cdot \eta .$$

Here, *P*_input_ is the incident optical power from the light source, *P*_trans_ is the power transmitted through the GNP suspension, and η is the photothermal conversion efficiency. The energy dissipated to the surrounding medium is defined as:


[3] $$Q_{\text{diss}}=\left(T_{\text{max}}-T_{\text{0}}\right)\cdot hS,$$

where *S* is the surface area of the cuvette exposed to the surrounding environment, *h* is the heat transfer coefficient, *T*_max_ is the maximum temperature attained on irradiating the suspension for time *t*, and *T*_0_ is temperature of the surrounding environment. By combining Equation 2 and Equation 3, the energy balance equation becomes


[4] $$\sum_{j}C_{j}m_{j}\frac{\text{d}T}{\text{d}t}=\left(P_{\text{input}}-P_{\text{trans}}\right)\cdot \eta -\left(T_{\max}-T_{0}\right)\cdot hS.$$

For a nanoparticle suspension, the specific heat capacity and total mass of GNPs are much smaller than those of water and can be neglected. Thus, by only considering the specific heat capacity of water (*C*_w_ = 4184 J·kg^−1^·K^−1^) and the total mass of water (*m*_w_) the energy balance equation becomes:


[5] $$C_{\text{w}}m_{\text{w}}\frac{\text{d}T}{\text{d}t}=\left(P_{\text{input}}-P_{\text{trans}}\right)\cdot \eta -\left(T_{\max}-T_{0}\right)\cdot hS.$$

The change in temperature with time is thus:


[6] $$\frac{\text{d}T}{\text{d}t}=\frac{\left(P_{\text{input}}-P_{\text{trans}}\right)\cdot \eta }{C_{\text{w}}m_{\text{w}}}-\frac{\left(T_{\text{max}}-T_{\text{0}}\right)\cdot hS}{C_{\text{w}}m_{\text{w}}}.$$

Here, 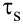 = (*hS*)/(*C*_w_*m*_w_) can be considered as the rate constant associated with the heat loss from the nanoparticle suspension. This rate constant can be determined from the slope of temperature decay profile of the suspension after the light source is turned off. At thermal equilibrium, the temporal change in the temperature becomes almost zero. Hence, the photothermal conversion efficiency can be described as:


[7] $$\eta =\frac{\left(T_{\text{max}}-T_{\text{0}}\right)}{\left(P_{\text{input}}-P_{\text{trans}}\right)}-m_{\text{w}}C_{w}\tau_{\text{s}}.$$

By using Equation 6 and Equation 7, the temperature rise of a GNP suspension under irradiation is described as:


[8] $$T\left(t\right)=T_{0}+\frac{\left(P_{\text{input}}-P_{\text{trans}}\right)}{m_{\text{w}}C_{\text{w}}\tau_{\text{s}}}\cdot \eta \cdot \left(1-\exp\left(-\tau_{\text{s}}t\right)\right).$$

Equation 7 and Equation 8 can be used to determine the amount of heat absorption, the photothermal conversion efficiency, or the temperature rise of the GNP suspension during photothermal interaction.

## Results and Discussion

### Spectral absorbance of GNPs

**Figure 4:**
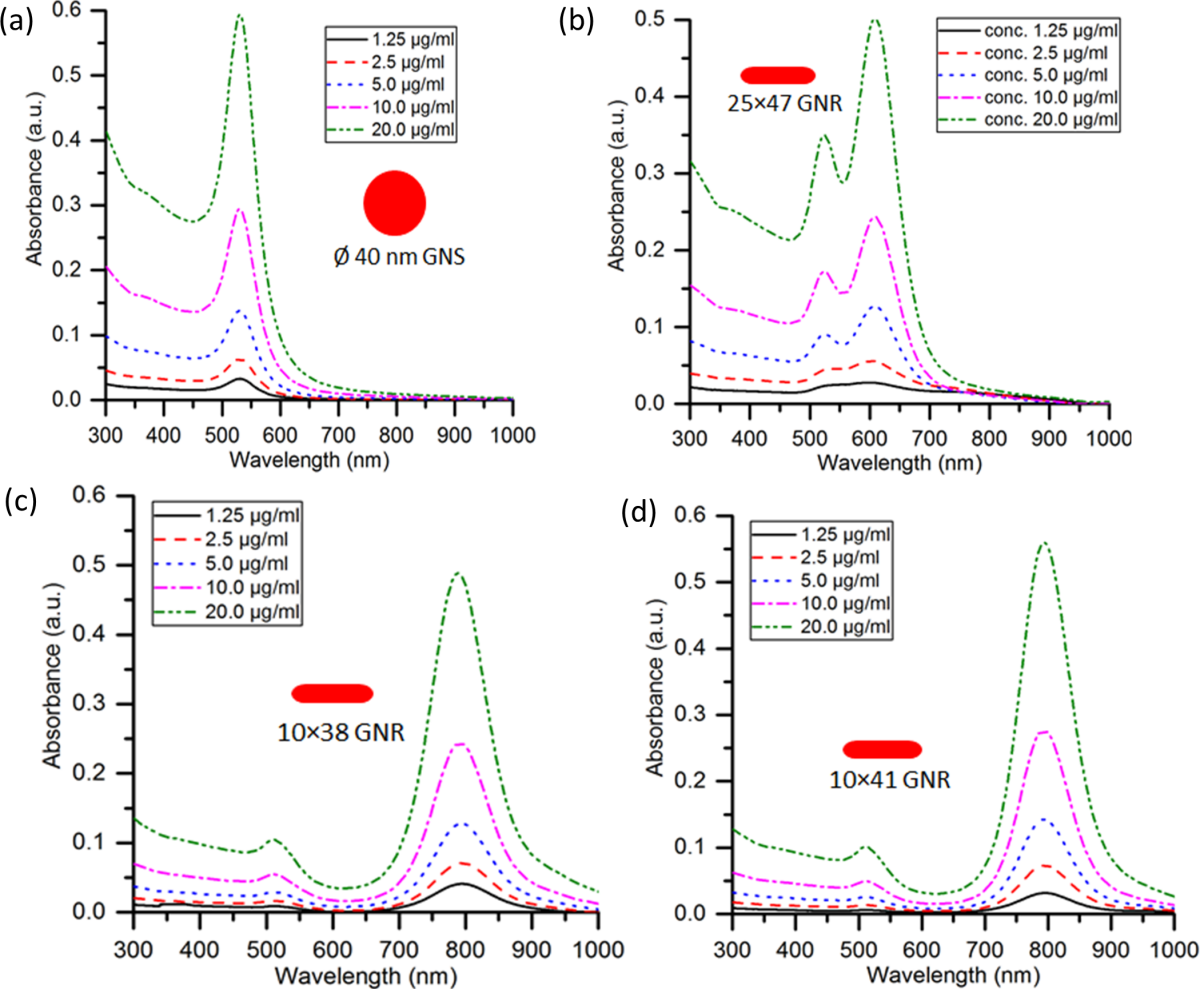
Spectral absorbance of different concentrations of nanoparticles. (a) 40 nm GNSs, (b) 25 × 47 nm GNRs, (c) 10 × 38 nm GNRs, and (d) 10 × 41 nm GNRs.

The concentration-dependent spectral absorbance of GNSs of 40 nm diameter and GNRs with sizes of 25 × 47 nm, 10 × 38 nm, and 10 × 41 nm measured by using a UV–vis spectrophotometer (UV-3200, Labindia Instruments Pvt. Ltd.) is shown in Figure 4. The measured peak absorbance wavelength of the GNPs is in agreement with the localized surface plasmon resonance (LSPR) reported for these batches by the suppliers (Table S1, Supporting Information File 1). Figure 4a shows that the LSPR of GNSs is at 530 nm. Figure 4b–d shows that the GNRs show two resonance peaks, that is, a first peak at 520–525 nm (transverse mode) for all GNRs and a second peak (longitudinal mode) at 600 nm for GNRs of 25 × 47 nm, at 790 nm for GNRs of 10 × 38 nm, and at 800 nm for GNRs of 10 × 41 nm size, respectively.

### Absorbed optical power by GNPs

**Figure 5:**
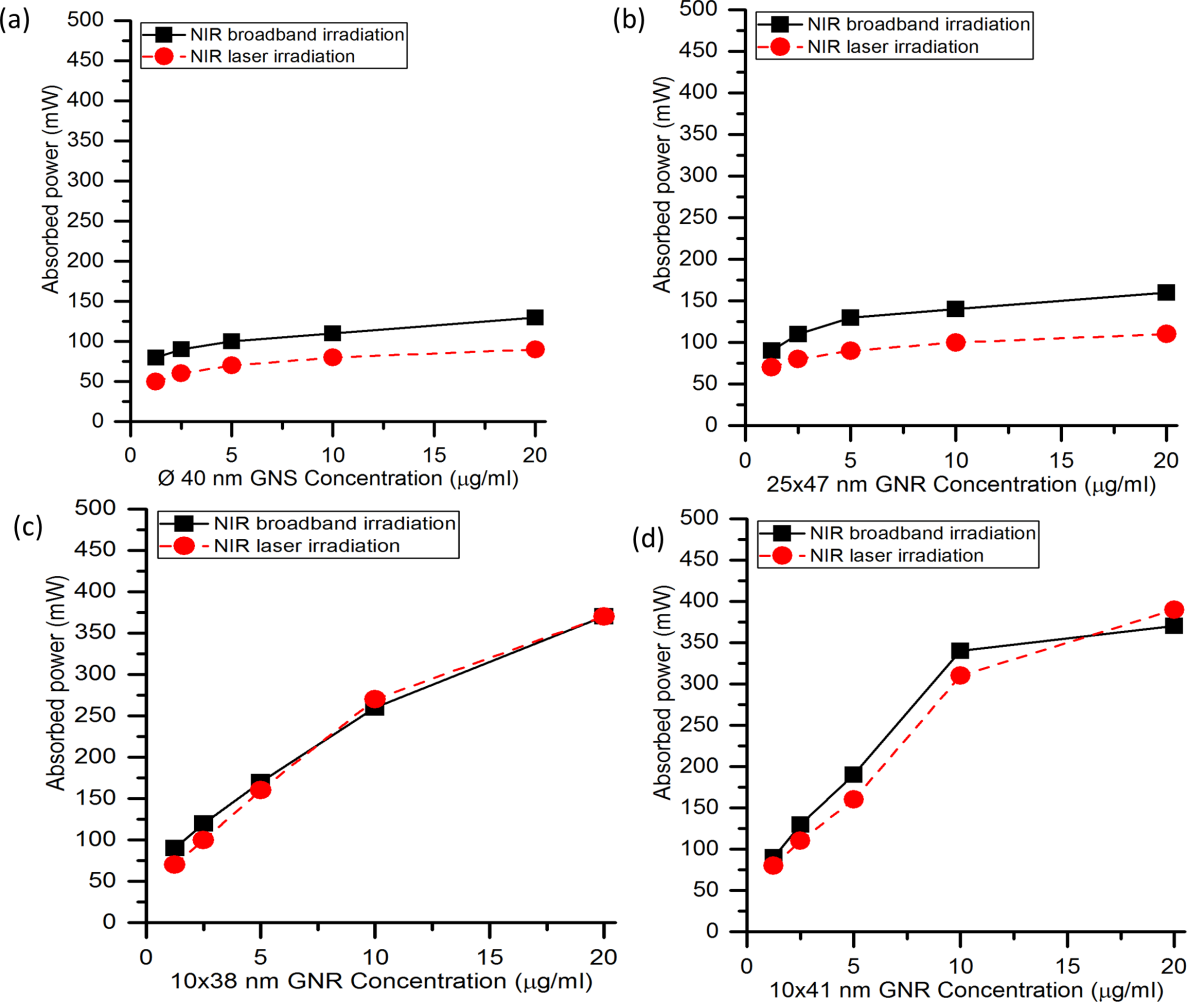
Concentration-dependent variation of the absorbed optical power for (a) 40 nm GNSs, (b) 25 × 47 nm GNRs, (c) 10 × 38 nm GNRs, and (d) 10 × 41 nm GNRs under NIR broadband irradiation and NIR laser irradiation. The incident optical power is 500 mW.

The optical power absorbed by GNPs enhances the temperature of the medium. The concentration-dependent variation of the absorbed optical power, measured for 40 nm GNSs, 25 × 47 nm GNRs, 10 × 38 nm GNRs, and 10 × 41 nm GNRs under NIR broadband irradiation and NIR laser irradiation with irradiation power of 500 mW (irradiation intensity ca. 0.8 W/cm^2^ over a beam diameter of 9 mm) is shown in Figure 5.

Figure 5a shows that on increasing the nanoparticle concentration from 1.25 to 20 µg/mL, the absorbed optical power for GNSs increases from 80 to 130 mW under NIR broadband irradiation and from 50 to 85 mW under NIR laser irradiation. Similarly, Figure 5b shows that the absorbed optical power for 25 × 47 nm GNRs increases from 90 to 160 mW under NIR broadband irradiation and from 70 to 105 mW under NIR laser irradiation. Figure 5c and Figure 5d show, respectively, that the absorbed power for 10 × 38 nm GNRs increases from 90 to 370 mW under NIR broadband irradiation and from 70 to 370 mW under NIR laser irradiation. For 10 × 41 nm GNRs, it increases from 90 to 370 mW and from 80 to 390 mW under NIR broadband and laser irradiation, respectively.

The optical power absorbed by 10 × 38 nm and 10 × 41 nm GNRs is almost equal under NIR laser and NIR broadband irradiation. Also, it is higher than that of 40 nm GNSs and 25 × 47 nm GNRs because of the match of between the plasmonic wavelengths and the source wavelength. Upon increasing the concentration of GNPs, a nonlinear behavior of absorbed power with respect to the concentration of GNPs was observed. This is because with an increase in the concentration of GNPs, the scattering increases in relation to the absorption [30]. Also, with an increase in the concentration of GNPs, the absorption of the incident radiation occurs predominantly in the first few layers of the suspension [31], and there may be interparticle coupling of plasmon reponses, which can be looked at in future studies. Further, it can be observed that NIR broadband irradiation is more suitable for heat generation when the plasmonic wavelength of the nanoparticles differs considerably from the wavelength of the laser source.

### Temperature rise of GNP suspensions on photothermal interaction

**Figure 6:**
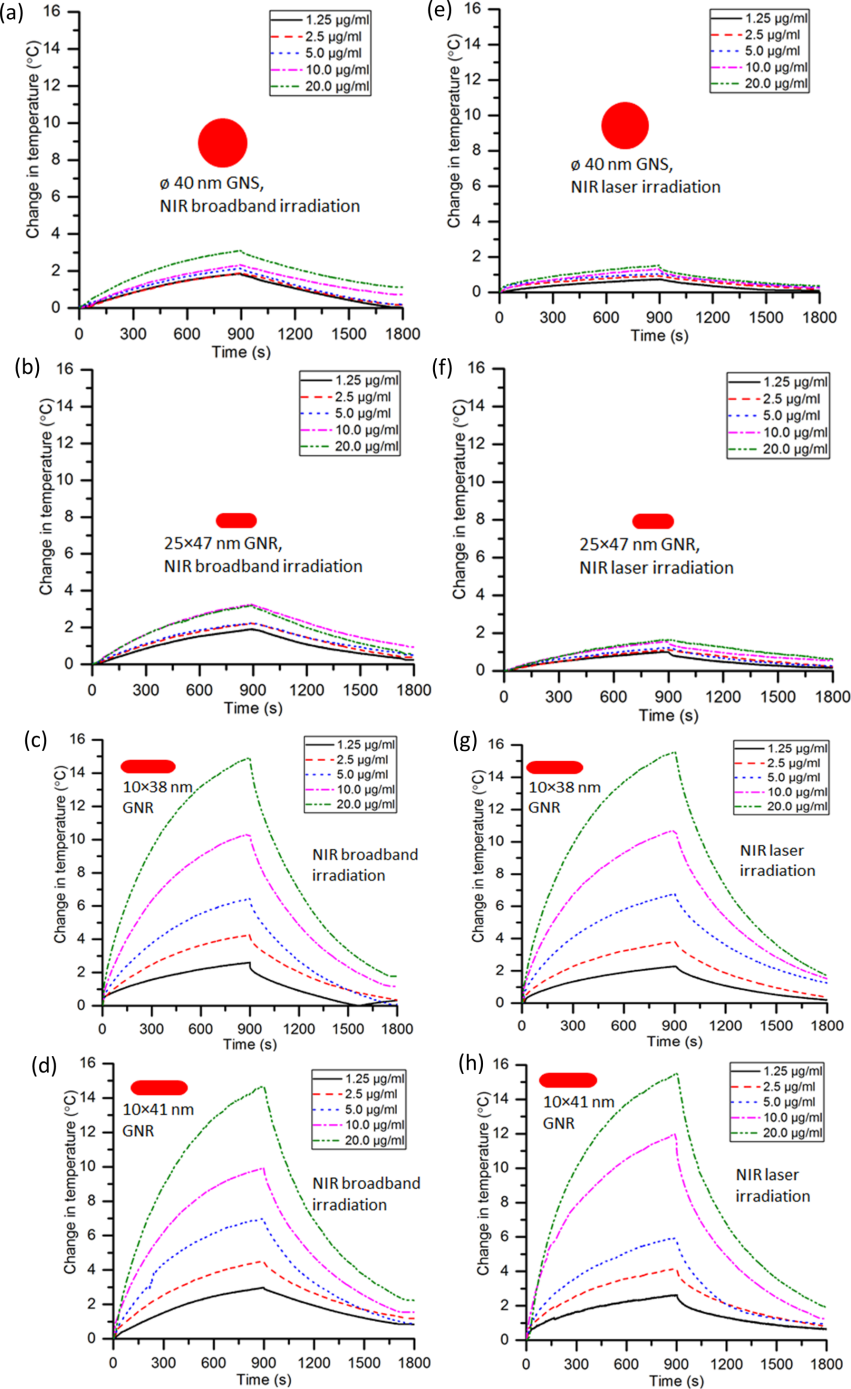
Temporal variation of the suspension temperature (heating and cooling) for different nanoparticle concentrations of (a) 40 nm GNSs, (b) 25 × 47 nm GNRs, (c) 10 × 38 nm GNRs, and (d) 10 × 41 nm GNRs under NIR broadband irradiation and (e) 40 nm GNSs, (f) 25 × 47 nm GNRs, (g) 10 × 38 nm GNRs, and (h) 10 × 41 nm GNRs under NIR laser irradiation. The temperatures were measured for 900 s of each irradiation (heating) and cooling.

The temporal change in the temperature of deionized (DI) water when the NIR broadband and laser irradiation was on (heating) and off (cooling) over a period of 1800 s is shown in Figure S2 (Supporting Information File 1). The concentration-dependent temperature of the GNP suspensions measured over a period of 1800 s is shown in Figure 6. Figure 6a and Figure 6e show that the maximum change in temperature for GNSs is 3.0 and 1.5 °C for NIR broadband and NIR laser irradiation, respectively. It increases, respectively, by 1.2 and 0.8 °C on increasing the GNS concentration from 1.25 to 20 µg/mL. For GNSs it can be seen that the temperature increases ca. 100% more on NIR broadband irradiation than on NIR laser irradiation.

It is seen from Figure 6b and Figure 6f that for 20 µg/mL of 25 × 47 nm GNRs, the increases in temperature are 3.1 and 1.6 °C under NIR broadband and NIR laser irradiation, respectively. The temperature rise is increased by 1.2 and 0.6 °C under NIR broadband and NIR laser irradiation, respectively, when the concentration is increased from 1.25 to 20 µg/mL. The overall temperature rise for 25 × 47 nm GNRs is about 94% higher under NIR broadband irradiation than under NIR laser irradiation.

Figure 6c and Figure 6g show that the maximum changes in temperature obtained for 10 × 38 nm GNRs of 20 µg/mL concentration are 14.9 and 15.6 °C for NIR broadband and NIR laser irradiation, respectively. The temperature rise is enhanced by 12.5 and 13.3 °C under NIR broadband and NIR laser irradiation, respectively, when the concentration is increased from 1.25 to 20 µg/mL. Because of the good match between LSPR and laser wavelength, these GNRs show a 4% higher temperature rise under NIR laser irradiation compared to broadband irradiation.

It is seen from Figure 6d and Figure 6h that the maximum temperature rises for 10 × 41 nm GNRs are, respectively, 14.7 and 15.5 °C under NIR broadband and laser irradiation, which is ca. 5% more under NIR laser irradiation than under broadband irradiation. On increasing the concentration from 1.25 to 20 µg/mL, the temperature change is enhanced by 11.7 and 12.9 °C under NIR broadband and NIR laser irradiation, respectively.

Overall, the temperature rise for 10 × 38 nm and 10 × 41 nm GNRs is higher under NIR laser irradiation than under to broadband irradiation, while GNSs and 25 × 47 nm GNRs yielded a higher temperature rise under NIR broadband irradiation. Overall, the temperature measurements show similar trends, for 25 × 47 nm GNRs, 10 × 38 nm GNRs, and 10 × 41 nm GNRs, that is, the temperature increases rapidly for about 500 s and then rises more slowly up to 900 s for concentrations below 10 µg/mL. While 10 × 38 nm GNRs and 10 × 41 nm GNRs show a rapid increase in temperature for concentrations greater than or equal 10 µg/mL under irradiation. In contrast, the temperature of GNSs with a concentration below 10 µg/mL hardly rises after 250 s. Because of the match between plasmonic wavelength and incident irradiation, 10 × 38 nm GNRs and 10 × 41 nm GNRs show a temperature increase for a longer period of time than GNSs and 25 × 47 nm GNRs. The photothermal stability of the GNPs was examined by measuring the spectral absorbance of GNPs before and after irradiation as shown in Figure S3 (Supporting Information File 1). It is seen that there is no change in the optical absorption of the nanoparticles and that they are stable.

### Photothermal conversion efficiency of GNPs

**Figure 7:**
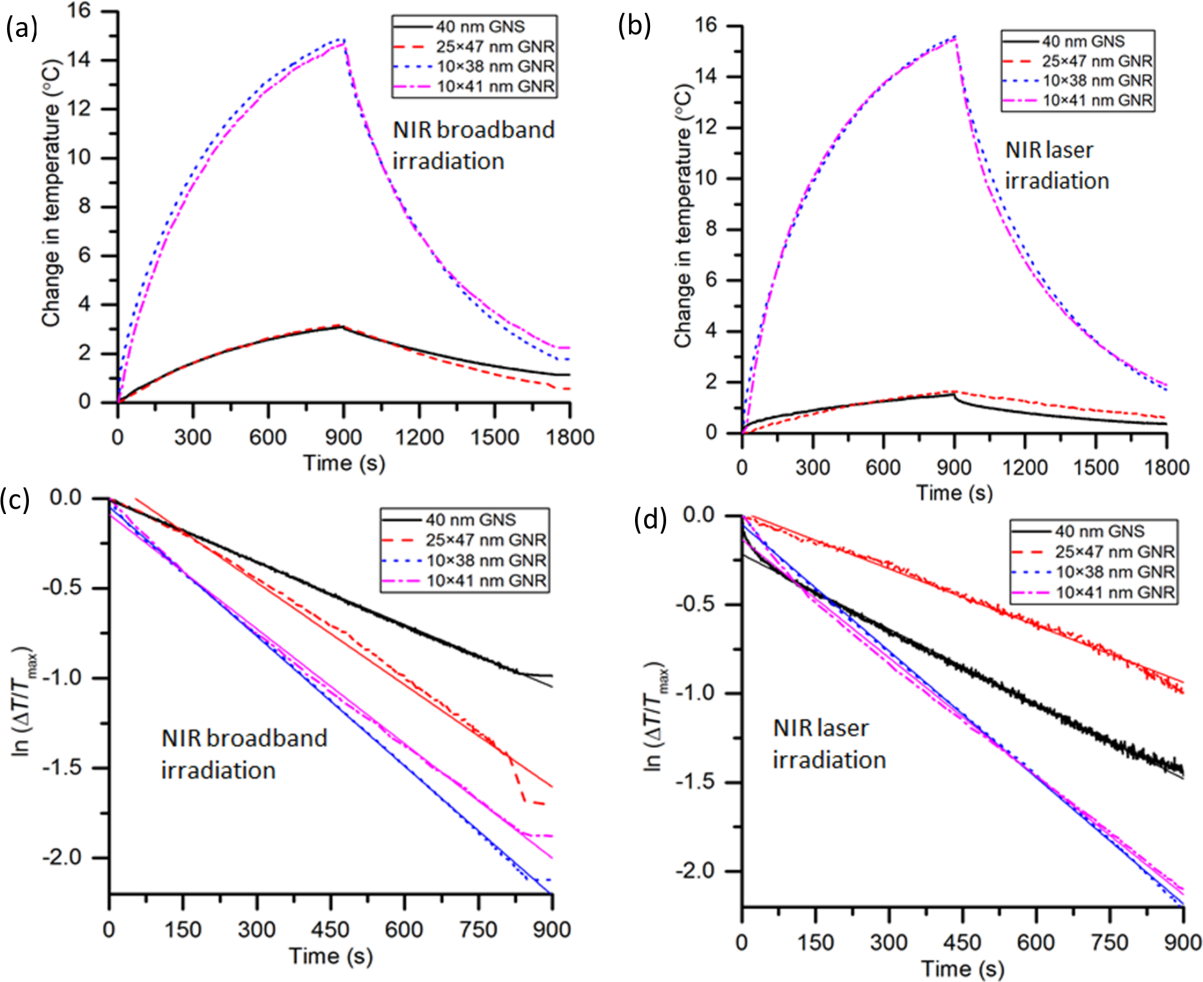
Heating and cooling temperature profiles for 40 nm GNSs, 25 × 47 nm GNRs, 10 × 38 nm GNRs, and 10 × 41 nm GNRs of 20 µg/mL concentration under (a) NIR broadband irradiation and (b) NIR laser irradiation. Corresponding curves of ln(∆*T*/*T*_max_) to calculate the rate constant of heat loss for (c) NIR broadband irradiation and (d) NIR laser irradiation.

The photothermal conversion efficiency (*η*) of the GNPs was evaluated based on the obtained heating and cooling temperature profiles. The measured temperature profiles for GNPs of 20 µg/mL concentration under NIR broadband and laser irradiation are shown in Figure 7a and Figure 7b, respectively.

From Figure 7a, it is seen that 10 × 38 nm GNRs and 10 × 41 nm GNRs yield almost equal maximum temperature rises of 14.9 and 14.7 °C under NIR broadband irradiation. Similarly, from Figure 7b, it is observed that under NIR laser irradiation, 10 × 38 nm GNRs and 10 × 41 nm GNRs yielded a maximum temperature increase of ca. 15.5 °C within 900 s, while there is an exponential decay of temperature after the light source is switched off. For GNSs and 25 × 47 nm GNRs, NIR broadband irradiation resulted in a slightly higher temperature rise.

From Equation 7, it is inferred that the photothermal conversion efficiency of GNPs depends on the maximum attained temperature as well as the rate of heat loss. Here, the rate of heat loss can be determined by calculating the slope of ln(∆*T*/*T*_max_) as a function of the time during cooling. Figure 7c and Figure 7d show the plots of ln(∆*T*/*T*_max_) as a function of the time for GNPs of 20 µg/mL concentration under NIR broadband and NIR laser irradiation, respectively. The slope is determined by a linear fit. The calculated slope and *R*^2^ values are given in Table 1.

**Table 1:** Calculated slope and *R*^2^ values for different GNPs under NIR broadband and laser irradiation.

Parameter/GNPs	40 nm GNS	25 × 47 nm GNR	10 × 38 nm GNR	10 × 41 nm GNR

slope *m* (s^−1^) (broadband irradiation)	0.0014	0.00107	0.00237	0.00222
slope *m* (s^−1^) (laser irradiation)	0.00115	0.0019	0.0024	0.00213
*R*^2^ (equal values for broadband and laser)	0.99539	0.9936	0.9995	0.99656

Table 1 shows *R*^2^ values of more than 0.99, representing adequate fits. Also, it is seen that under NIR broadband and laser irradiation, the rate constants of heat loss for 10 × 38 nm GNRs and 10 × 41 nm GNRs are higher than those of GNSs and 25 × 47 nm GNRs.

**Figure 8:**
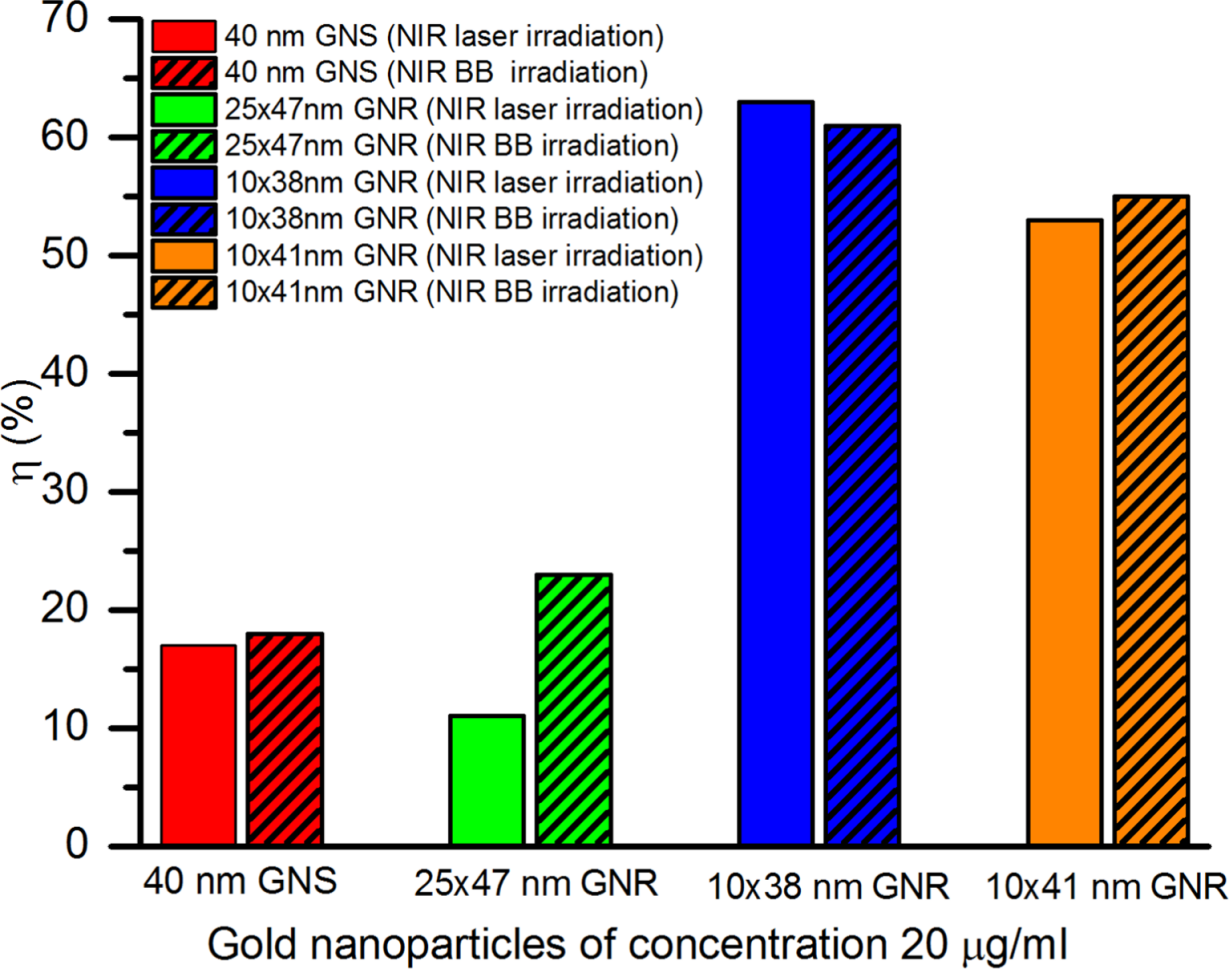
Photothermal conversion efficiency of 40 nm GNSs, 25 × 47 nm GNRs, 10 × 38 nm GNRs, and 10 × 41 nm GNRs of 20 µg/mL concentration under NIR broadband and NIR laser irradiation.

Based on the measured values of maximum temperature rise, absorbed power, specific heat capacity (*C*_w_ = 4184 J·kg^−1^·K^−1^), and total mass (*m*_w_ = 1.495 g) of the suspensions, the calculated photothermal conversion efficiency (*η*) of the different GNPs under NIR broadband (BB) and laser irradiation are shown in Figure 8. The efficiency ranges within 17–63% for the different GNPs.

Figure 8 shows that for NIR laser and NIR broadband irradiation, respectively, the photothermal conversion efficiencies of 40 nm GNSs are 17% and 18%, for 25 × 47 nm GNRs are 11% and 23%, for 10 × 38 nm GNRs are 63% and 61%, and for 10 × 41 nm GNRs are 53% and 55%. Also, 40 nm GNSs, 25 × 47 nm GNRs, and 10 × 41 nm GNRs show a 6%, 110% and 4% higher photothermal conversion efficiency under NIR broadband irradiation than under NIR laser irradiation. The photothermal efficiency of 10 × 38 nm GNRs is 3% higher under NIR laser irradiation than under NIR broadband irradiation. These results show that the heat generation of GNPs highly depends on size and shape of the GNPs as well as on the incident wavelength. In general, GNRs with the surface plasmon response matching the irradiation wavelength exhibit maximum photothermal conversion efficiency under both laser or broadband irradiation. Broadband irradiation results in a much higher efficiency for 25 × 47 nm GNRs, whose peak absorption wavelength highly differs from the irradiation wavelength.

A photothermal conversion efficiency of 53% for 10 × 41 nm gold nanorods, as determined in our study, has also been reported by Cole and co-workers [8]. Similarly, reported efficiencies within 51–95% for GNRs of varying aspect ratios between 2.8 and 3.8 under irradiation with an 808 nm CW laser match with our calculated values [21].

### Concentration-dependent photothermal conversion efficiency

**Figure 9:**
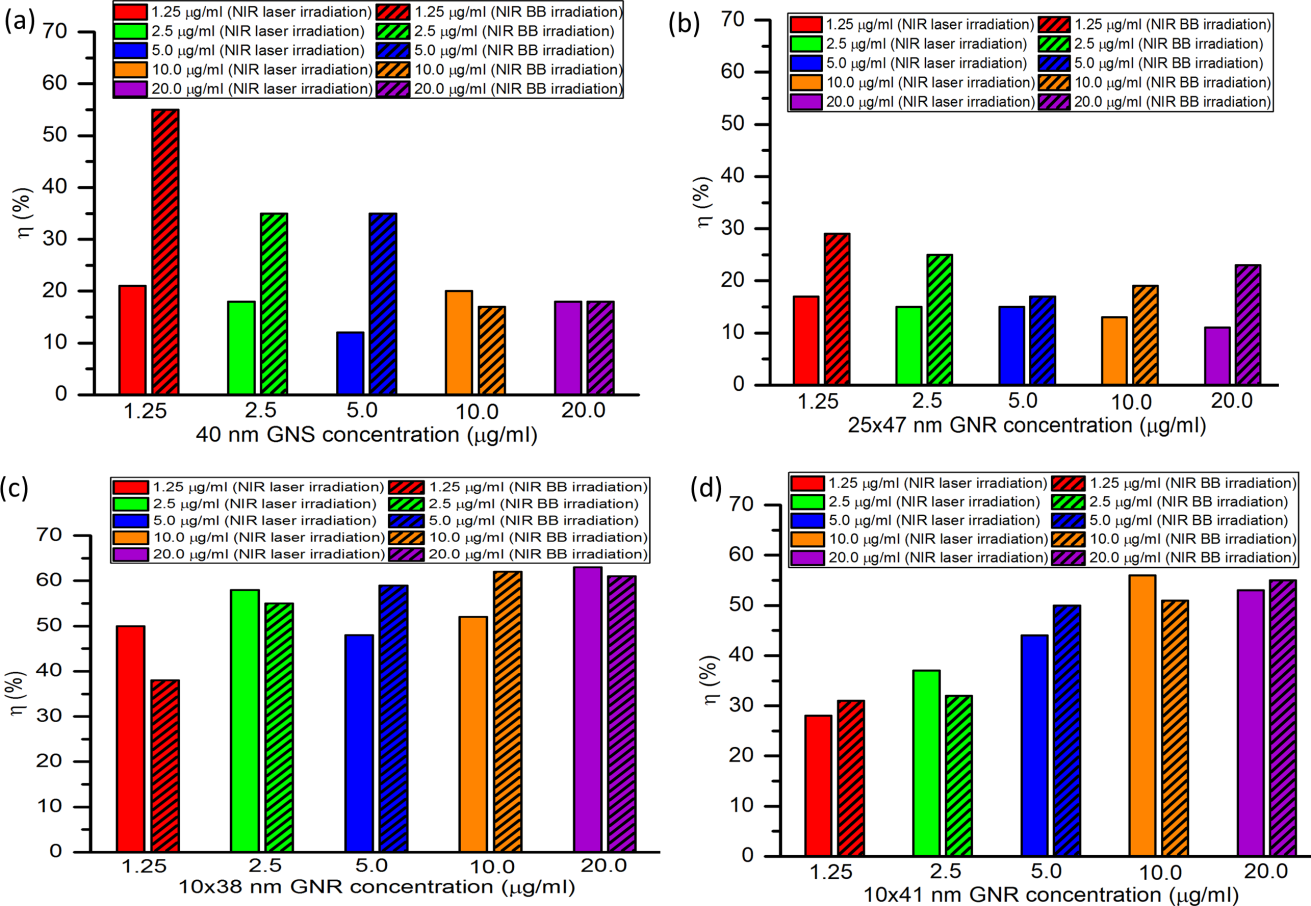
Photothermal conversion efficiency of different nanoparticle concentrations for (a) 40 nm GNSs, (b) 25 × 47 nm GNRs, (c) 10 × 38 nm GNRs, and (d) 10 × 41 nm GNRs under NIR broadband and NIR laser irradiation.

The concentration-dependent photothermal conversion efficiency of GNPs is shown in Figure 9. The concentration-dependent plots of ln(∆*T*/*T*_max_) as a function of time are shown in Supporting Information File 1, Figure S1.

From Figure 9a, it is seen that for GNS concentrations from 1.25 to 5 µg/mL, the photothermal conversion efficiency decreases from 21% to 12%, while for GNS concentrations from 5 to 20 µg/mL, the efficiency increases from 12% to 20% under NIR laser irradiation. Under NIR broadband irradiation, the photothermal conversion efficiency of GNSs decreases from 55% to 18% on increasing the GNS concentration from 1.25 to 20 µg/mL. Overall, for GNS concentrations of 1.25 to 5 µg/mL, the photothermal conversion efficiency of GNSs is higher under NIR broadband irradiation than under NIR laser irradiation, while for GNS concentrations of 10 and 20 µg/mL, the efficiency is lower under NIR broadband irradiation or almost equal.

For 25 × 47 nm GNRs, on increasing the concentration from 1.25 to 20 µg/mL, the photothermal conversion efficiency decreases from 17% to 11% under NIR laser irradiation as shown in Figure 9b. Under NIR broadband irradiation, the efficiency of GNRs decreases from 29% to 17% and from 17% to 23% on increasing the concentration from 1.25 to 5 µg/mL and from 5 to 20 µg/mL, respectively. Overall, for 25 × 47 nm GNRs, the photothermal conversion efficiency is higher under NIR broadband irradiation than under NIR laser irradiation. Also, the photothermal conversion efficiency of GNSs is higher than that of 25 × 47 nm GNRs under NIR laser and broadband irradiation.

Figure 9c shows that under NIR laser irradiation, the photothermal conversion efficiencies of 10 × 38 nm GNRs are 50% and 58% for concentrations of 1.25 and 2.5 µg/mL, respectively, while the efficiency decreases from 58% to 48% on increasing the concentration from 2.5 to 5 µg/mL. At a further increase in GNR concentration from 5 to 20 µg/mL, the efficiency increases from 48% to 63%. Under NIR broadband irradiation, the photothermal conversion efficiency of 10 × 38 nm GNRs increases from 38% to 62% for an increase in concentration from 1.25 to 20 µg/mL. Also, the efficiency of 10 × 38 nm GNRs shows higher values under NIR laser irradiation than under NIR broadband irradiation for concentrations of 1.25, 2.5, and 20.0 µg/mL, while for concentrations of 5.0 and 10.0 µg/mL, NIR broadband irradiation yields a higher efficiency. The concentration-dependent photothermal conversion efficiency of 10 × 38 nm GNRs is higher under NIR laser and broadband irradiation than that of GNSs and 25 × 47 nm GNRs because of the better match between incident wavelength and the SPR peak of the GNRs.

Figure 9d shows that, under NIR laser irradiation, the photothermal conversion efficiency of 10 × 41 nm GNRs increases from 28% to 56% with an increase in concentration from 1.25 to 10.0 µg/mL. At an increase in concentration from 10 to 20 µg/mL, there is a slight decrease in the efficiency from 56% to 53%. Whereas, under NIR broadband irradiation, the photothermal conversion efficiency of 10 × 41 nm GNRs increases from 31% to 55% for an increase in concentration from 1.25 to 20 µg/mL. The efficiency of 10 × 41 nm GNRs at different concentrations shows higher values than those of GNSs and 25 × 47 nm GNRs, while the efficiency values of 10 × 41 nm GNRs are lower than the efficiencies of 10 × 38 nm GNRs.

Overall, the photothermal conversion efficiencies of 10 × 38 nm and 10 × 41 nm GNRs are higher than those of GNSs and 25 × 47 nm GNRs. For low concentrations, GNSs show high efficiency under NIR broadband irradiation, while 10 × 38 nm GNRs and 10 × 41 nm GNRs show higher photothermal conversion efficiency at higher concentrations for both NIR broadband and NIR laser irradiation.

### Photothermal conversion efficiencies for different irradiation powers

**Figure 10:**
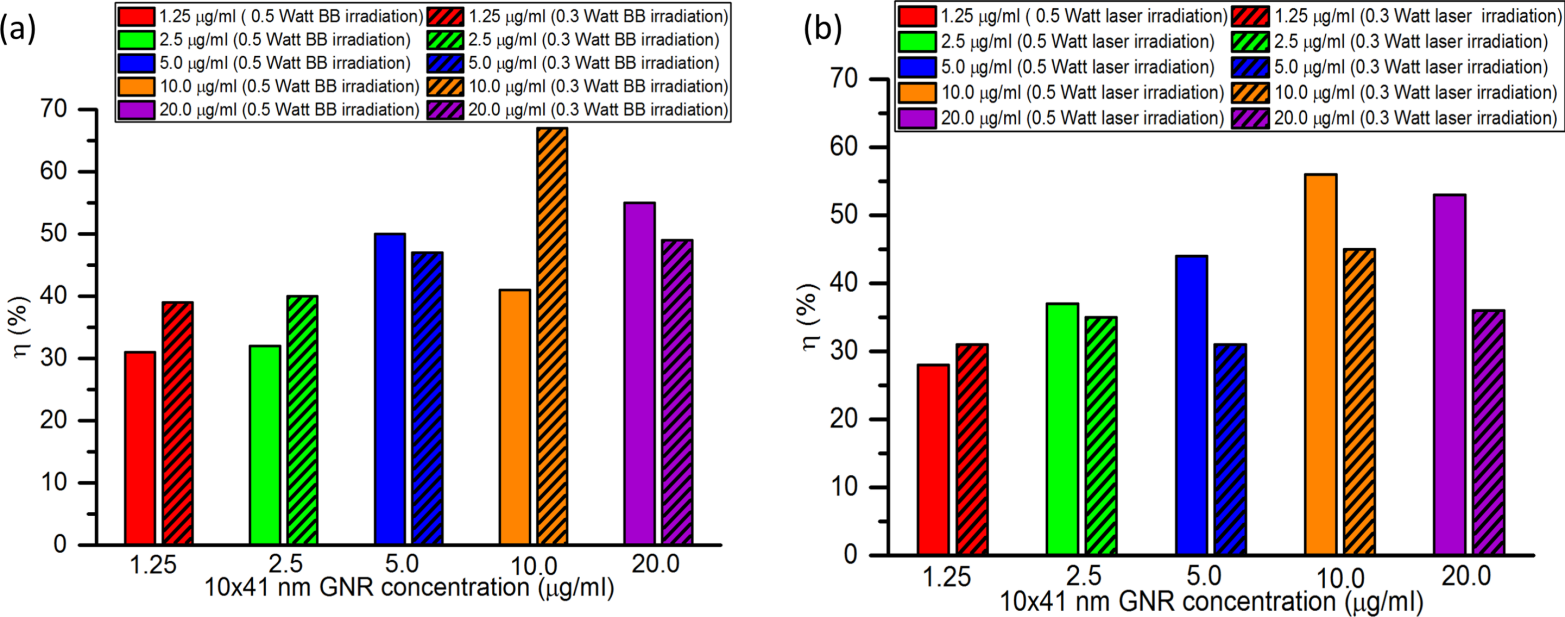
Concentration-dependent photothermal conversion efficiency of 10 × 41 nm GNRs irradiated with optical powers of 0.5 and 0.3 W under (a) NIR broadband irradiation and (b) NIR laser irradiation.

The same light sources were used with different irradiation powers to see the effect of optical power on the photothermal conversion efficiency. The concentration-dependent photothermal conversion efficiencies of 10 × 41 nm GNRs under NIR laser and broadband irradiation for irradiation powers of 0.5 and 0.3 W are shown in Figure 10a and Figure 10b, respectively.

From Figure 10a, it is seen that under NIR broadband irradiation, the photothermal conversion efficiency of 10 × 41 nm GNRs for concentrations of 1.25, 2.5, and 10.0 µg/mL is, respectively, 8%, 8%, and 26% higher for an irradiation power of 0.3 W than for 0.5 W. While for concentrations of 5.0 and 20.0 µg/mL, the efficiency is 3% and 6% lower for 0.3 W. Overall, under broadband irradiation, the lower irradiation power results in similar or higher photothermal conversion efficiencies for the different nanoparticle concentrations.

Under NIR laser irradiation, as seen from Figure 10b, the photothermal conversion efficiency is 3% higher for a concentration of 1.25 µg/mL, and 2% to 17% lower for concentrations between 2.5 and 20.0 µg/mL for an irradiation power of 0.3 W as compared to 0.5 W. Overall, under NIR laser irradiation, the photothermal conversion efficiency increases with an increase in optical power. An earlier study by Alrahili et al. also reported a higher photothermal efficiency of 81% for 10 × 41 nm GNRs using a higher incident power of 4 W.

## Conclusion

The concentration-dependent photothermal conversion efficiencies, under NIR laser (808 nm) and NIR broadband irradiation (754–816 nm), of GNSs with 40 nm diameter, 25 × 47 nm GNRs, 10 × 38 nm GNRs, and 10 × 41 nm GNRs have been evaluated. 40 nm GNSs, 25 × 47 nm GNRs, and 10 × 41 nm GNRs show, respectively, 6%, 110%, and 4% higher efficiencies under NIR broadband irradiation than under NIR laser irradiation, while 10 × 38 nm GNRs show almost equal efficiencies under laser and broadband irradiation.

For 40 nm GNSs under NIR broadband irradiation, on increasing the concentration from 1.25 to 20.0 µg/mL, there is a decrease in photothermal conversion efficiency, while under NIR laser irradiation, the efficiency reaches a minimum value at about 5 µg/mL and increases for other concentrations. For 25 × 47 nm GNRs, under NIR laser irradiation, the efficiency decreases slightly for higher concentrations, but under broadband irradiation, the efficiency shows a dip at a concentration value of 5 µg/mL and increases for lower and higher concentrations.

The higher efficiency for 25 × 47 nm GNRs implies that broadband irradiation seems suitable to attain higher efficiency for the nanoparticles whose peak absorption wavelength is differs strongly from the irradiation wavelength. Lower concentrations (1.25–5 µg/mL) of GNSs and 25 × 47 nm GNRs yield 2–3 times higher efficiencies under NIR broadband irradiation. For 10 × 38 nm GNRs and 10 × 41 nm GNRs, the different concentrations show almost equal efficiencies for NIR laser and broadband irradiation. On increasing the irradiation power from 0.3 to 0.5 W, for 10 × 41 nm GNRs in a concentration range of 2.5–20.0 µg/mL, NIR laser irradiation results in 5% to 32% higher efficiencies, while NIR broadband irradiation leads to an increase by 6% to 11% in efficiency. Under broadband irradiation, a lower irradiation power results in almost equal or higher photothermal conversion efficiencies for different nanoparticle concentrations. Under NIR laser irradiation, the photothermal conversion efficiency increases with an increase in optical power. The reported maximum temperature increase of 16 °C corresponds to about 53 °C with respect to the physiological temperature of 37 °C, which is sufficient for thermal threatment of cancerous cells [32]. The discussed results may be useful for the selection of nanoparticle concentrations as well as irradiation sources and irradiation power for a variety of applications involving the plasmonic photothermal phenomenon. Further, the variation in plasmonic wavelengths of gold nanomaterials embedded in a medium, for instance, in tissue during plasmonic photothermal therapeutics, can be accomodated through the use of a NIR broadband source.

## Supporting Information

File 1 Additional experiemental data.
