# Supplementary material for: Concentration-dependent photothermal conversion efficiency of gold nanoparticles under near-infrared laser and broadband irradiation
Source: Beilstein J Nanotechnol. 2023 Feb 6;14:205–17. doi: 10.3762/bjnano.14.20 (PMC9924363; doi:10.3762/bjnano.14.20)
Supplement: File 1 — Additional experiemental data. [file Beilstein_J_Nanotechnol-14-205-s001.pdf]

BEILSTEIN JOURNAL OF NANOTECHNOLOGY

Supporting Information

for

Concentration-dependent photothermal conversion efficiency of gold nanoparticles under near-infrared laser and broadband irradiation

Vikas, Raj Kumar and Sanjeev Soni

Beilstein J. Nanotechnol. **2023,** *14,* 205–217. doi:10.3762/bjnano.14.20

Additional experiemental data

License and Terms: This is a supporting information file under the terms of the Creative Commons Attribution License (https://creativecommons.org/licenses/by/4.0). Please note that the reuse, redistribution and reproduction in particular requires that the author(s) and source are credited and that individual graphics may be subject to special legal provisions.
The license is subject to the *Beilstein Journal of Nanotechnology* terms and conditions: (https://www.beilstein-journals.org/bjnano/terms)

Table S1: Different GNPs used in this study.

GNPs type	Size (nm)	Peak absorption wavelength (nm)	Standard Concentration (µg/ml)	Product no.	Procured from
Gold nanosphere (GNS)	40	529–533	45	741981	Sigma Aldrich, USA
Gold nanorods (GNR)	25×47	600	45	771651	Sigma Aldrich, USA
GNR	10×38	790	38.5	A12-10-780-CTAB-DIH-1-25	Nanopartz, USA
GNR	10×41	806	39	A12-10-808-CTAB-DIH-1-25	Nanopartz, USA

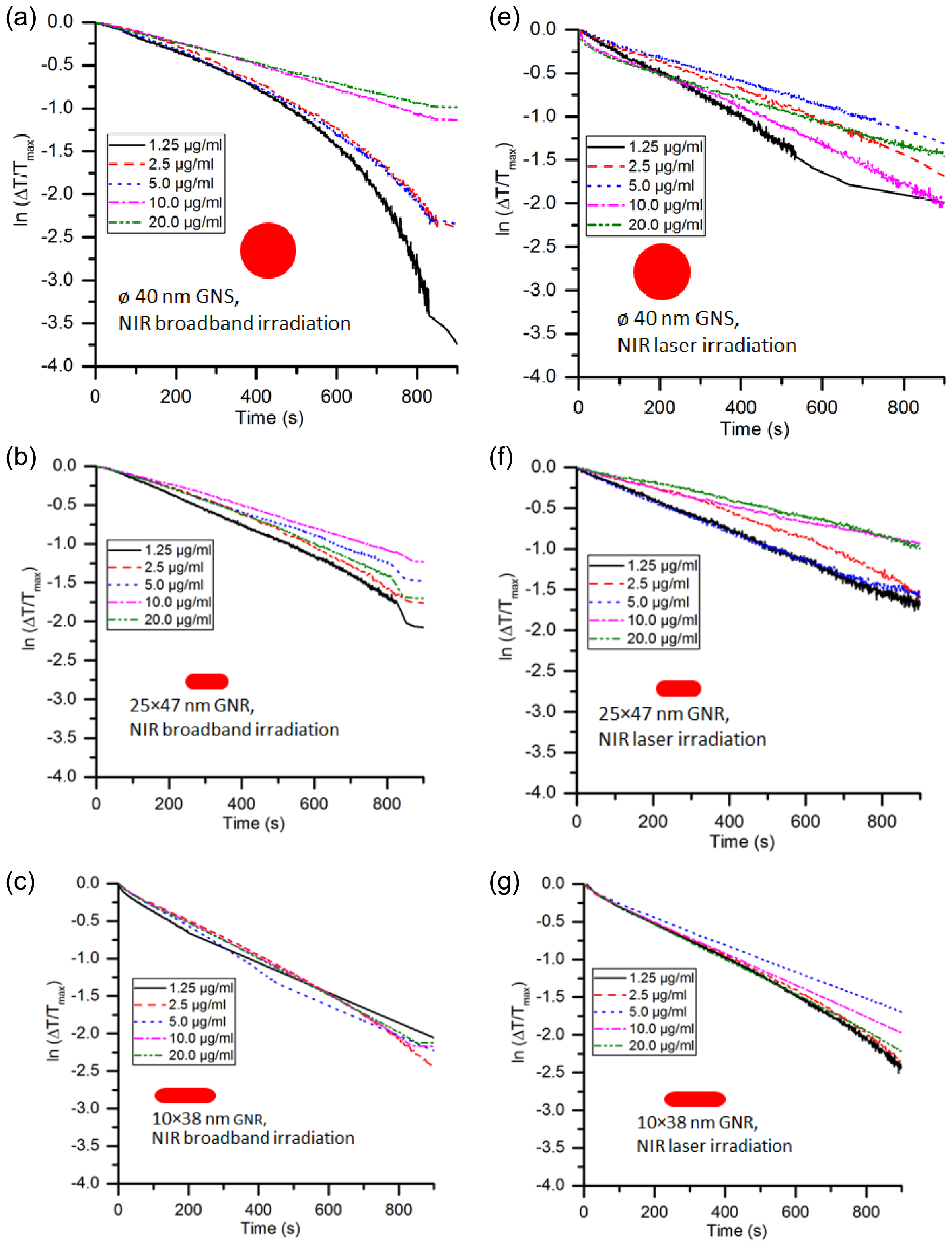
(a)
ln (ΔT/T_max)
Time (s)
1.25 μg/ml
2.5 μg/ml
5.0 μg/ml
10.0 μg/ml
20.0 μg/ml
ø 40 nm GNS,
NIR broadband irradiation
(b)
25×47 nm GNR,
NIR broadband irradiation
(c)
10×38 nm GNR,
NIR broadband irradiation
(e)
ø 40 nm GNS,
NIR laser irradiation
(f)
25×47 nm GNR,
NIR laser irradiation
(g)
10×38 nm GNR,
NIR laser irradiation

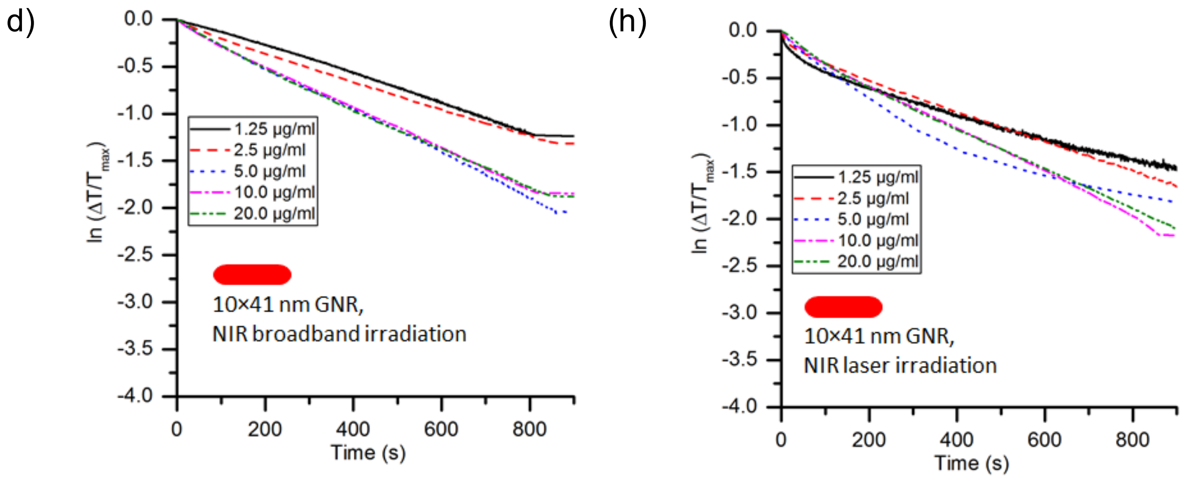

Figure S1: Temporal variation of ln($\Delta T/T_{max}$) for calculating the rate constant of heat loss of different nanoparticle concentrations for (a) 40 nm GNSs, (b) 25 × 47 nm GNRs, (c) 10 × 38 nm GNRs, and (d) 10 × 41 nm GNRs under NIR broadband irradiation (754–816 nm), and (e) 40 nm GNSs, (f) 25 × 47 nm GNRs, (g) 10 × 38 nm GNRs, and (h) 10 × 41 nm GNRs under NIR laser irradiation (808 nm).

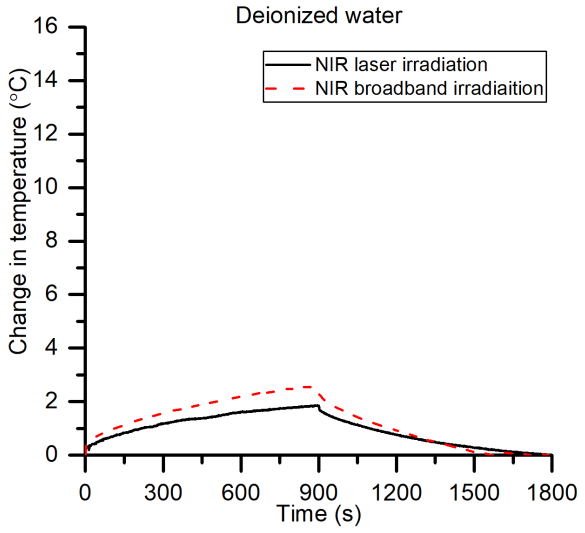

Figure S2: Heating and cooling profiles of deionized water under NIR broadband and NIR laser irradiation.

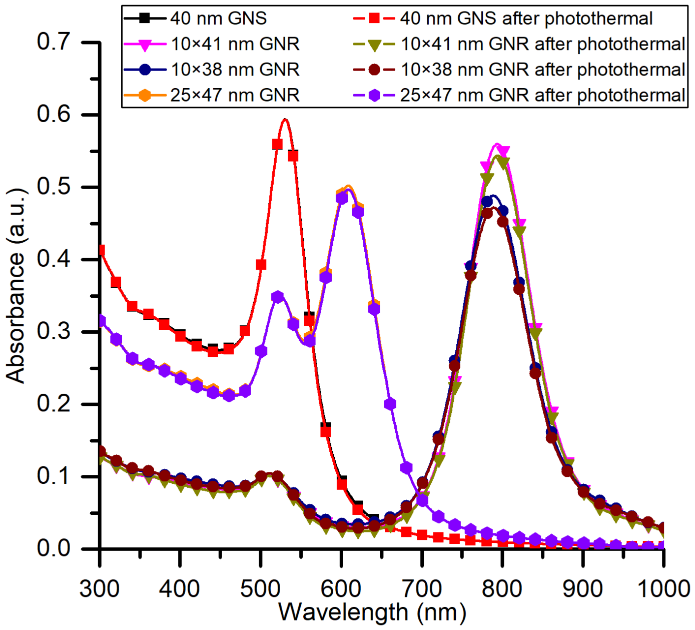

Figure S3: Spectral absorbance, measured using a spectrophotometer, of (a) 40 nm GNSs, (b) 25 × 47 nm GNRs, (c) 10 × 38 nm GNRs, and (d) 10 × 41 nm GNRs before and after irradiation.
